# The role of insulin and incretin-based drugs in biliary tract cancer: epidemiological and experimental evidence

**DOI:** 10.1007/s12672-022-00536-8

**Published:** 2022-08-07

**Authors:** Hua Sun, Xiaohui Qi

**Affiliations:** 1 Department of Geriatrics, Zhejiang Hospital of Integrated Traditional Chinese and Western Medicine, No.208 East Huancheng Road, Hangzhou, Zhejiang China; 2 Department of Endocrine and Metabolic Diseases, Shanghai Institute of Endocrine and Metabolic Diseases, Ruijin Hospital, Shanghai Jiao Tong University School of Medicine, No.197 Ruijin Er Road, Shanghai, China; 3 Shanghai National Clinical Research Center for Metabolic Diseases, Key Laboratory for Endocrine and Metabolic Diseases of the National Health Commission of the PR China, Shanghai Key Laboratory for Endocrine Tumor, State Key Laboratory of Medical Genomics, Ruijin Hospital, Shanghai Jiao Tong University School of Medicine, No.573 Xujiahui Road, Shanghai, China

**Keywords:** Insulin, Incretin, Antidiabetic, Biliary tract cancer, Cholangiocarcinoma

## Abstract

Insulin and incretin-based drugs are important antidiabetic agents with complex effects on cell growth and metabolism. Emerging evidence shows that insulin and incretin-based drugs are associated with altered risk of biliary tract cancer (BTC). Observational study reveals that insulin is associated with an increased risk of extrahepatic cholangiocarcinoma (ECC), but not intrahepatic cholangiocarcinoma (ICC) or gallbladder cancer (GBC). This type-specific effect can be partly explained by the cell of origin and heterogeneous genome landscape of the three subtypes of BTC. Similar to insulin, incretin-based drugs also exhibit very interesting contradictions and inconsistencies in response to different cancer phenotypes, including BTC. Both epidemiological and experimental evidence suggests that incretin-based drugs can be a promoter of some cancers and an inhibitor of others. It is now more apparent that this type of drugs has a broader range of physiological effects on the body, including regulation of endoplasmic reticulum stress, autophagy, metabolic reprogramming, and gene expression. In particular, dipeptidyl peptidase-4 inhibitors (DPP-4i) have a more complex effect on cancer due to the multi-functional nature of DPP-4. DPP-4 exerts both catalytic and non-enzymatic functions to regulate metabolic homeostasis, immune reaction, cell migration, and proliferation. In this review, we collate the epidemiological and experimental evidence regarding the effect of these two classes of drugs on BTC to provide valuable information.

## Introduction

Biliary tract cancer (BTC) consists of intrahepatic cholangiocarcinoma (ICC), extrahepatic cholangiocarcinoma (ECC), and gallbladder cancer (GBC) [1, 2]. BTC is rare but lethal and prevalent in some areas all around the world, such as several countries in Asia, Eastern Europe, and Latin America [3, 4]. Several risk factors are already established for BTC which include parasitic infections, primary sclerosing cholangitis (PSC), choledochal cyst, hepatolithiasis, and toxins. Other potential risk factors include inflammatory bowel disease (IBD), hepatitis B virus (HBV), hepatitis C virus (HCV), cirrhosis, diabetes, obesity, and smoking [5–7]. Among them, close attention should be paid to diabetes mellitus (DM) because of its rapid growth worldwide today.

According to the latest report from the International Diabetes Federation, the global diabetes prevalence in 20 ~ 79-year-olds in 2021 was estimated to be 10.5% (536.6 million people), rising to 12.2% (783.2 million) in 2045 [8]. More importantly, as a chronic and progressive metabolic disease, DM needs to be treated with antidiabetic drugs to maintain stable blood glucose levels and avoid the serious consequences of long-term hyperglycemia. However, in addition to their hypoglycemic effects, antidiabetic drugs may have several side effects, including an increased risk of certain types of cancer.

Among hypoglycemic agents, insulin and incretin-based drugs are two important drugs that are widely used in clinical practice. Considering their complex regulatory role in cell growth and metabolism, the influence of insulin and incretin-based drugs on the cancer profile of diabetic patients has attracted widespread attention. In the past few years, many studies have shown that insulin and incretin-based drugs are associated with an increased risk of multiple cancer types, such as pancreatic cancer, breast cancer, and thyroid cancer [9–14]. Recently, some important findings related to insulin/incretin-based drugs and BTC have been reported, which provide new evidence for further study. A study from China showed that insulin was associated with an increased risk of ECC but not ICC or GBC [15], indicating a type-specific relationship between insulin and BTC. A comprehensive meta-analysis of randomized clinical trials showed that incretin-based drugs were not associated with an increased risk of BTC [16]. This result is inconsistent with previous findings which have shown that incretin-based drugs played a facilitative role in the development of BTC [17].

The limited and inconsistent results pose a challenge to the clarification of the effect of insulin and incretin-based drugs on BTC. To facilitate research progress in this area, here we update the epidemiological and experimental evidence on the association of insulin and incretin-based drugs with BTC risk and explore the involving molecular mechanisms.

## Insulin and BTC: limited evidence from only one cohort study

### The mitogenic effect of insulin and its molecular mechanisms

**Fig. 1:**
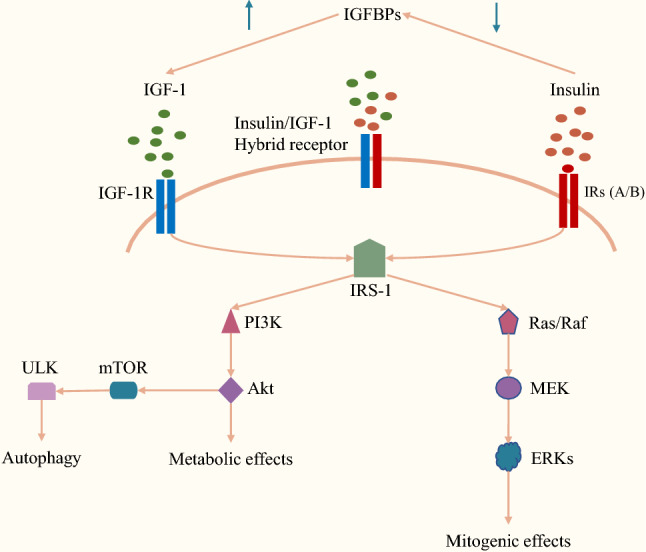
Insulin signaling and crosstalk with the IGF-1 systems. Insulin regulates the Ras/Raf/MAPK and PI3K/AKT/mTOR pathways to influence the development of BTC. In the condition of hyperinsulinemia, insulin can directly bind to IGF-1R and IR/IGF-1Rs or indirectly increase the biologically active free IGF-1 to augment its mitogenic and antiapoptotic effects. * IGF-I* insulin-like growth factor-1, *MAPK* mitogen-activated protein kinase, *PI3K* phosphatidylinositol-3-kinase, *Akt* protein kinase B, *mTOR* mammalian target of rapamycin, *BTC* biliary tract cancer, *IGF-1R* insulin-like growth factor-1 receptor, *IR/IGF-1Rs* hybrid receptors of insulin receptor and insulin-like growth factor-1 receptor, *IRS* insulin receptor substrates, *IRs(A/B)* insulin receptor A or B isoform, *IGFBPs* insulin-like growth factor-binding proteins, *ULK* Unc-51 like autophagy activating kinase, *MEK* mitogen-activated protein kinase kinase, *ERK* extracellular signal-regulated kinase

Since first identified by Canadian scientists Frederick G. Banting and Charles H in 1921, insulin has marked one of the most important breakthroughs in the history of diabetic treatment. Insulin is secreted by the beta cells of the islets of Langerhans in the pancreas to regulate the level of glucose in the blood. Upon binding to the insulin receptor (IR), insulin activates its tyrosine kinase and initiates downstream signaling including the phosphatidylinositol 3-kinase/protein kinase B (PI3K/Akt) [18–20], mammalian target of rapamycin (mTOR) [21–23], and Ras/mitogen-activated protein kinase (MAPK) pathways [20, 24, 25]. Noteworthy, besides the metabolic effect, insulin plays an important role in the regulation of cell growth and exerts a mitogenic effect on tumor development. Firstly, through the insulin receptor substrates (IRSs), an adaptor protein of the IR, the insulin signaling pathway recruits multiple signaling complexes to connect with other signaling pathways [26–29]. For instance, the recruiting of growth factor receptor-bound protein 2 (Grb2) to the binding motif on IRS forms a complex with guanine nucleotide exchange factor Son of Sevenless and phosphorylates Ras [30, 31], thus activating the MAPK signaling cascade [32, 33]. In addition, the upregulation of Rac1, another important signaling pathway downstream of the IR [34], promoted cell proliferation and migration as well as angiogenesis in multiple cancer types [34–38]. Moreover, insulin also promotes tumor cell growth by altering the energy metabolism of tumor cells through the aberrant mTOR pathway to upregulate glucose uptake [39] and glycogenolysis [22]. Finally, the signaling crosstalk between insulin and insulin-like growth factor-1 receptor (IGF-1R) is closely related to tumor development. Since insulin with 40–80% homology to the insulin-like growth factor-1 (IGF-1) [40] and IGF-1R with a 70% homology to the IR [41], in the condition of hyperinsulinemia, insulin can overcome the restriction of lower affinity to the IGF-1R and activate the downstream signaling of the IRS/PI3K/Akt and Ras/Raf/extracellular-signal-regulated kinase (ERK) pathways, thus augments its mitogenic and antiapoptotic effects [42–44]. In addition, hyperinsulinemia also exerts its proliferative effects indirectly by reducing the liver production of IGF-1-binding proteins [45] to increase the biologically active free IGF-1. Noticeably, increased insulin levels can also bind to the hybrid receptors IR/IGF-1Rs (HRs) [46] and activate the insulin/IGF signaling system (Fig. 1 shows the insulin signaling and crosstalk with the IGF-1 systems).

### Insulin and increased risk of BTC

Previous epidemiological studies indicated that insulin promotes higher rates of some cancer types presentation in patients with type 2 diabetes mellitus (T2DM), such as colorectal cancer [47, 48], lung cancer [49], breast cancer [50], pancreatic cancer [10, 51], liver cancer [52–54], bladder cancer [55] and neuroendocrine tumors [55]. In addition to the observational evidence, many experimental studies provided solid confirmation of the positive relationship between insulin and cancer. Saisana et al. reported that insulin has direct tumor-promoting effects on the gastric adenocarcinoma cells [56]. In the case of breast cancer, evidence revealed that insulin stimulates the proliferation of some human breast cancer cell lines in vitro by activating both the PI3K/Akt and the MAPK signaling pathways [57]. Although there have been many studies investigating the association between insulin and cancer, research on BTC is still very sparse. It is very challenging to conduct large-scale epidemiological investigations to clarify the relationship between insulin and BTC due to the relatively low incidence of BTC worldwide (0.3-6 per 100,000 inhabitants per year) [58, 59].

Back in 2013, Schlesinger and colleagues attempted to assess the risk of BTC in diabetic patients treated with insulin, but they were unable to conclude because only two cases of BTC were observed in 2,156 insulin users [60]. To fill this knowledge gap in this area, we conducted a study in 2021 using electronic medical record data, which showed that insulin therapy was associated with an increased hazard ratio (HR) of ECC (HR, 4.10; 95% CI, 1.54–10.92) but not ICC (HR, 1.36; 95% CI, 0.30–6.09) or GBC (HR, 1.28; 95% CI, 0.61–2.66) (Table 1) [15]. However, only this study has yielded some statistically significant results, and due to residual confounding factors, it is still unable to draw definitive conclusions about the relationship between insulin and BTC. More results from other studies are urgently needed to elucidate the effect of insulin on BTC in diabetic patients.

**Table 1:** Association of insulin and incretin-based drugs with BTC from epidemiological studies

Year	Drug	Cancer	Relative risk (95% CI)	Sample size	Country/Region	Reference
2022	Insulin	ECC	4.10, 1.54–10.92*	202,557	China	15
2022	Insulin	ICC	1.36, 0.30–6.09*	202,557	China	15
2022	Insulin	GBC	1.28, 0.61–2.66*	202,557	China	15
2015	DDP-4i	BTC	0.75, 0.30–1.76*	16,492	Worldwide	132
2018	DDP-4i	CCA	1.77, 1.04–3.01*	614,274	UK	17
2020	DDP-4i	CCA	0.98, 0.75–1.29#	744 cases, 2,976 controls	Italy	133
2021	DDP-4i	CCA	1.15, 0.90–1.46*	346,485	Scandinavian	134
2016	GLP-1-RA	BTC	1.62, 0.67 to 3.90*	9,340	Worldwide	132
2018	GLP-1-RA	CCA	1.97, 0.83–4.66*	614,274	UK	17
2020	GLP-1-RA	CCA	1.09, 0.63–1.89#	744 cases, 2,976 controls	Italy	133
2021	GLP-1-RA	CCA	1.25, 0.89–1.76*	239,391	Scandinavian	134
2022	GLP-1 RA	BTC	1.43, 0.80–2.56^	103,371	Worldwide	16

*BTC* biliary tract cancer,* ECC* extrahepatic cholangiocarcinoma,* ICC* intrahepatic cholangiocarcinoma,* GBC* gallbladder cancer,* CCA* cholangiocarcinoma (ECC and ICC),* CI* confidence interval,* DDP-4* dipeptidylpeptidase-4 inhibitor,* GLP-1 RA* glucagon-like peptide-1 receptor agonist,* UK* United Kingdom

* Hazard ratio; # Odds ratio; ^ Relative risk

### Possible reasons for insulin and BTC risk discrepancy

According to our previous findings, the association of insulin with BTC seems to be type-specific. Based on available evidence, the reasons for the type-specific nature lie in two aspects: different cells of origin (COO) and different molecular alterations.

**Fig. 2:**
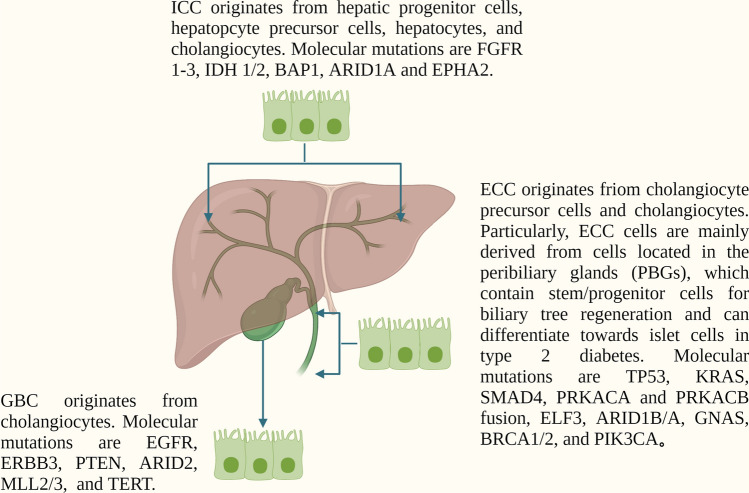
Cell of origin and molecular alterations of three subtypes of BTC. The difference in the cell of origin and molecular alterations lays the foundation of the risk discrepancy between insulin and three subtypes of BTC. Compared to ICC and GBC, ECC originates mainly from cells residing in PBGs and has more frequent mutations in P53, KRAS, and SMAD4, resulting in greater sensitivity to insulin stimulation. * BTC* biliary tract cancer, *ICC* intrahepatic cholangiocarcinoma, *ECC* extrahepatic cholangiocarcinoma, *GBC* gallbladder cancer, *PBGs* peribiliary glands, *FGFR 1–3* fibroblast growth factor receptor 1–3, *IDH 1/2* isocitrate dehydrogenase, *BAP1* BRCA1-associated-protein 1,ubiquitin carboxyl-terminal hydrolase, *ARID1A* AT-rich interactive domain-containing protein 1 A, *EPHA2* ephrin type-A receptor 2, *TP53* tumor protein P53, *KRAS* kirsten rat sarcoma viral oncogene homolog, *SMAD4* mothers against decapentaplegic homolog 4, *PRKACA* protein kinase cyclic adenosine monophosphate (cAMP)-activated catalytic subunit alpha, *PRKACB* protein kinase cAMP-activated catalytic subunit beta, *ELF3* E74 like ETS transcription factor 3, *ARID1B/A* AT-rich interactive domain-containing protein 1 B/A, *GNAS* guanine nucleotide-binding protein-alpha stimulating, *BRCA1/2* breast cancer gene 1/2, *PIK3CA* phosphatidylinositol-4,5-bisphosphate 3-kinase catalytic subunit alpha, *EGFR* epidermal growth factor receptor, *ERBB3* erythroblastic leukemia viral oncogene homologue3, *PTEN* phosphatase and tensin homolog, *ARID2* AT-rich interactive domain 2, *MLL2/3* histone-lysine N-methyltransferase, *TERT* telomerase reverse transcriptase. (Fig. 2 is created with BioRender.com)

Firstly, BTC consists of a heterogenetic group of cancers that originate from different cell types throughout the biliary tree (Fig. 2). ICC originates from hepatic progenitor cells, hepatocytes, or cholangiocytes, GBC originates from cholangiocytes, and ECC originates from cholangiocyte precursor cells and cholangiocytes [61]. The difference between COO lays the biological foundation for the risk discrepancy between subtypes of BTC. In particular, the COO of ECC chiefly originates from peribiliary glands (PBGs), which contain stem/progenitor cells (BTSCs) for the regeneration of the biliary tree and can differentiate toward pancreatic islet beta-cell in T2DM 62, 63]. This phenomenon indicates that when exposed to hyperinsulinemia and hyperglycemia for a prolonged period, the continuous stimulation will contribute to the proliferation of PBGs cells. In turn, both in vivo and in vitro evidence suggests that hyperinsulinemia can stimulate preneoplastic and tumor cells with genetic mutations to become clinically manifest tumors and more aggressive malignancies [64–66]. Therefore, the reason why insulin can significantly increase the risk of ECC may be that insulin is highly carcinogenic to the cells of PBGs.

Secondly, the molecular alterations of BTC subtypes differ based on different COO (Fig. 2). The frequent mutations in ICC are fibroblast growth factor receptor 1–3 (FGFR 1–3), isocitrate dehydrogenase (IDH 1/2), BRCA1-associated-protein 1 (BAP1, ubiquitin carboxyl-terminal hydrolase), AT-rich interactive domain-containing protein 1 A (ARID1A), and ephrin type-A receptor 2 (EPHA2), in ECC are tumor protein P53 (TP53), kirsten rat sarcoma viral oncogene homolog (KRAS), mothers against decapentaplegic homolog 4 (SMAD4), protein kinase cyclic adenosine monophosphate (cAMP)-activated catalytic subunit alpha (PRKACA), and protein kinase cAMP-activated catalytic subunit beta (PRKACB) fusion, E74 like ETS transcription factor 3 (ELF3), AT-rich interactive domain-containing protein 1 B/A (ARID1B/A), guanine nucleotide-binding protein-alpha stimulating (GNAS), breast cancer gene 1/2 (BRCA1/2), and phosphatidylinositol-4,5-bisphosphate 3-kinase catalytic subunit alpha (PIK3CA), and in GBC are epidermal growth factor receptor (EGFR), erythroblastic leukemia viral oncogene homologue 3 (ERBB3), phosphatase and tensin homolog (PTEN), AT-rich interactive domain 2 (ARID2), histone-lysine N-methyltransferase (MLL2/3), and telomerase reverse transcriptase (TERT) [67]Specifically, there are some mutations only occurring on a certain subtype of BTC. For example, IDH1/2 mutations and FGFR2 fusions frequently occur in ICC [68] and ERBB2 mutations frequently occur in GBC [69]. Subsequently, the various molecular alterations can lead to various deregulation of PI3K/Akt and MAPK pathways. Moreover, compared to ICC and GBC, ECC harbors more frequent mutations in the regulation of cell metabolism, growth, and proliferation, such as P53, KRAS, and SMAD4. These molecular mutations can form a complicated network with the insulin signaling pathway. For example, insulin can activate the effect of RAS [70] and interact with SMAD4 through some classical pathways, such as MAPK and PI3K/Akt [71, 72].

## Incretin-based drugs and BTC: inconsistency and conflicts

### The function of incretin-based drugs and cancer development

#### Incretin-based drugs and insulin regulation

Glucagon-like peptide-1 (GLP-1) is an incretin hormone secreted by enteroendocrine L cells in the distal intestine, alpha cells in the pancreas, and the central nervous system [73]. Since its discovery research began in 1979, GLP-1 has been recognized as an important stimulator of insulin secretion and a crucial regulator of energy homeostasis [74]. GLP-1 protects against hyperglycemia mainly by two mechanisms: enhancement of insulin secretion in beta-cells and inhibition of glucagon secretion in alpha-cells. In addition to immediately increasing the synthesis and release of insulin, its role in promoting insulin secretion by increasing proliferation and decreasing apoptosis of beta-cells at the late stage is particularly striking [75–78].

**Fig. 3:**
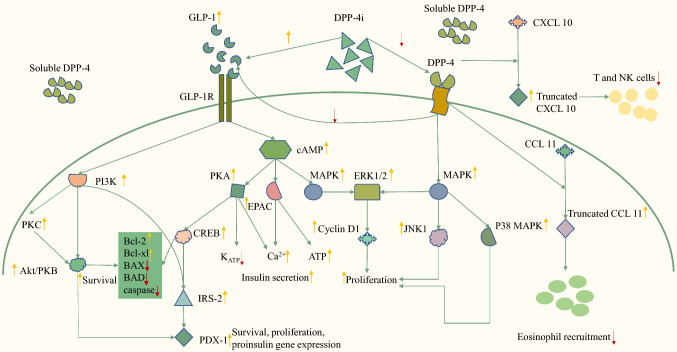
Signaling pathways of GLP-1 and DPP-4. GLP-1 elicits its incretin effects via an acute elevation in cAMP levels and subsequent activation of PKA and EPAC when binding to GLP-1 receptors on beta-cells. GLP-1 slows the progressive loss of beta-cell function by the activation of pro-survival CREB signaling. This figure shows two of the common ways by which DPP-4 promotes cancer progression: truncating CCL11 to decrease the migration of eosinophils and CXCL10 to inhibit the migration of T and NK cells. * GLP-1* glucagon-like peptide-1, *DPP-4* dipeptidyl peptidase-4, *cAMP* cyclic adenosine monophosphate, *PKA* protein kinase A, *PKC* Protein kinase C, *Akt/PKB* Protein kinase B, *EPAC* exchange protein directly activated by cAMP, *CREB* cAMP-response element binding protein, *NK* natural killer cells, *DPP-4i* dipeptidyl peptidase-4 inhibitors, *MAPK* mitogen-activated protein kinase, *PI3K* phosphatidylinositol-3-kinase, *mTOR* mammalian target of rapamycin, *JNK* c-Jun N-terminal kinase, *ERK1/2* extracellular signal-regulated kinase 1/2, *ATP* adenosine triphosphate, *IRS* insulin receptor substrates, *PDX-1* pancreatic and duodenal homeobox 1, *CCL* C-C motif chemokine ligand, *CXCL* chemokine (C-X-C motif) ligand

Firstly, as one of the most important substances in glucose-induced insulin secretion (GIIS), GLP-1 elicits its incretin effects via an acute elevation in cAMP levels, and subsequent activation of protein kinase A (PKA) and exchange protein directly activated by cAMP 2 (EPAC2) when binding to GLP-1 receptors on beta-cells (Fig. 3). Activation of PKA by cAMP results in phosphorylation of the sulphonylurea receptor (SUR1, a K_ATP_ channel subunit) [79] and subsequent membrane depolarization, then triggering the insulin secretory pathway. Activation of another cAMP effector, EPAC2, promotes membrane depolarization in lower concentrations of adenosine triphosphate (ATP) and subsequently stimulates insulin granule exocytosis and maturation by sensitizing the ryanodine receptors and activating the calcium-sensing complex [80–82].

Secondly, in addition to the acute stimulation of insulin secretion in response to a rise in blood glucose, GLP-1 slows the progressive loss of beta-cell function. By the activation of pro-survival cAMP-response element binding protein (CREB) signaling and non-receptor tyrosine kinase/c-Src and the transactivation of EGFR [83–85], GLP-1 induces increased expression of anti-apoptotic genes [84], attenuation of endoplasmic reticulum (ER) stress [86], prevention of oxidative stress and fatty acid-mediated toxicity [87], and subsequently exerts pro-survival and anti-apoptotic effects. Moreover, GLP-1 has been shown to alleviate glucotoxicity, lipotoxicity, excess nitric oxide (NO), and Ca^2+^ depletion in both primary beta-cells and cell lines to restore the insulin-secreting function of the pancreas [88–92].

Despite its important role in regulating insulin secretion, GLP-1 is very short-lived due to the degradation by a ubiquitous protease, dipeptidyl peptidase-4 (DPP-4), within one to two minutes under physiological conditions [93]. In order to prolong the half-life of GLP-1 and thus improve its clinical efficacy, several GLP-1 analogs (GLP-1 receptor agonists, GLP-1 RAs) have been developed. The half-life of these analogs, including exenatide, lixisenatide, liraglutide, dulaglutide, albiglutide, and semaglutide, can be extended to 2–13 h [94, 95], or up to 5 days [96]. Except the prolonged half-life, GLP-1 RAs share common mechanisms of action with the endogenous GLP-1: augmentation of hyperglycemia-induced insulin secretion, suppression of glucagon secretion at hyper- or euglycemia, and restoring the function of beta-cells [97–100]. Besides GLP-1 RAs, another widely used type of incretin-based drug is the DPP-4 inhibitor (DPP-4i). DPP4-i itself has no hypoglycemic activity. Instead, their hypoglycemic effect is mainly achieved by increasing endogenous GLP-1 concentrations [101], and their effects are more multifaceted, as described later.

#### GLP-1 RAs and cancer

At present, some preliminary and observational studies reported a link between GLP-1 RAs and cancers. Evidence showed that the GLP-1 receptors are not only expressed in normal tissues but also in malignant cells, such as endometrial cancer cells [102], breast cancer cells [94], colon cancer cells [103], and prostate cancer cells [104]. Considering its special effect on the pancreas, the relationship between GLP-1 RAs and pancreatic diseases attracted the attention of researchers. For instance, a case-control study reported that the use of GLP-1 RAs was associated with an increased risk of acute pancreatitis [105]. This raised the concern of increased risk of pancreatic cancer because recurrent acute pancreatitis turns into chronic pancreatitis and subsequently causes stenosis of the pancreatic duct and an increase in intra-ductal pressure, which leads to the development of pancreatic cancer over the years [106]. Actually, there is evidence that GLP-1 RAs are significantly associated with an increased risk of pancreatic cancer. The mechanism by which GLP-1 RAs exert their proliferative effect is still under exploration. Evidence showed that signaling pathways involved in the proliferative activity of GLP-1 are PI3-K/Akt, MAPK/ERK, and protein kinase C (PKC) pathways (Fig. 3) [107]. Another underlying mechanism is the incretin effect of GLP-1RAs. Since GLP-1RAs can elevate insulin levels both by increasing the immediate release of insulin and promoting the proliferation of beta-cells, concerns raise because of the tumorigenic effect of insulin as mentioned above.

#### DPP-4i and cancer

Before discussing the relationship between DPP-4i and cancer, it is necessary to introduce the pleiotropic effect of DPP-4 because the relationship between DPP-4i and cancer is the reverse of the relationship between DPP-4 and cancer. Besides its degradation effect on GLP-1, DPP-4 is also known as the T-cell antigen cluster of differentiation 26 (CD26) [108, 109]. It is a multi-functional protein widely expressed in different organs and on the surface of various cell types [110, 111]. DPP-4 is an integral membrane protein and consists of a large extracellular domain, anchored in the cell membrane by a flexible segment coupled to a trans-membrane sequence. It can exert catalytic functions with the C-terminal region of the extracellular part as well as non-enzymatic functions with the cysteine-rich region and glycosylation site-rich region [112].

When it exerts catalytic functions, DPP-4 cleaves incretin hormones related to metabolic homeostasis and a large number of cytokines, chemokines, and peptide hormones involved in the regulation of the immune system, such as GLP-1, glucose-dependent insulinotropic polypeptide (GIP), chemokine (C-X-C motif) ligand 10 (CXCL 10), CXCL 11, CXCL12, C-C motif chemokine ligand 2 (CCL2), CCL3, CCL5, CCL11, and CCL22 [113–115]. The catalytic effect of DPP-4 is reported to be associated with cancer progression. Evidence from hepatocellular carcinoma (HCC) showed that both gene ablation and pharmacological inhibition of DPP-4 notably prevent tumor progression by down-regulating the production of chemokine CCL2 [103]. In the investigation of HCC and breast cancer using syngeneic mouse models, Hollande and colleagues found that inhibition of DPP-4 increased the migration of eosinophils into solid tumors and reduced tumor growth. Further analysis showed that increased concentrations of the eosinophil chemoattractant CCL11, a known target for DPP4-mediated truncation, attributed to this antitumor effect (Fig. 3) [116]. Consistently, studies on melanoma and colorectal cancer also showed that DPP-4 limited the migration of T cells and natural killer (NK) cells by mediating the cleavage of chemokines CXCL10 to promote cancer progression (Fig. 3) [117, 118]. This effect can be attenuated by its inhibitor, sitagliptin, which can improve naturally occurring tumor immunity and enhance responses to T-cell-mediated immunotherapy.

Independent of its catalytic activity, DPP-4 can interact with other proteins as well as function as binding sites to affect physiological processes, such as the interaction between cells and the extracellular matrix, cell migration, and proliferation [112]. Evidence showed that DPP-4 overexpression led to increased epidermal growth factor-induced colony formation and expression of peptidylprolyl cis/trans-isomerase-1 (PIN1) and cyclin D1 in cancer cells via MEK/ERK and c-Jun N-terminal kinase (JNK) pathway, thus causing epithelial-mesenchymal transition (EMT) and tumorigenesis in breast cancer [119]. A study on endometrial carcinoma showed a positive association between DPP-4 expression and cancer cell proliferation, invasion, and tumorigenicity. Whereas, the proliferation of cancer cells was reduced when DPP-4 was knocked out or the enzyme activity was inhibited [120]. Moreover, within the immune system, DPP-4/CD26 can act as co-stimulatory molecules to amplify the immune signal, thereby leading to T-cell activation [121]. Consequently, the altered expression and/or activity of DPP-4 result in the disturbance of several pathological processes, including inflammation, viral entry, immune-mediated diseases, and tumor development [122–124]. When blocked by its inhibitor DPP-4i, the effect of DPP-4 on cancer will be attenuated or reversed.

### Incretin-based drugs and BTC

Since its debut in 2005, the incretin-based drug has achieved great success in the management of T2DM and obesity. In the investigation of the side effects of incretin-based drugs, several observational studies showed that the use of incretin-based drugs was associated with an increased risk of some cancer types, such as pancreatic cancer [13], breast cancer [125], and thyroid cancer [12, 126]. However, as the study progressed, more and more contradictions are emerging. For example, Liu and colleagues found that liraglutide treatment accelerated human triple-negative breast cancer (TNBC) cells’ progress both in vitro and in vivo through the nicotinamide adenine dinucleotide phosphate oxidase 4/reactive oxygen species/ vascular endothelial growth factor (NOX4/ROS/VEGF) signaling pathway [125]. On the contrary, Alanteet et al. showed opposite results. In their study, liraglutide caused 48% inhibition of MCF-7 human breast cancer cell proliferation in obese adipose tissue-derived stem cells-conditioned medium, reduced the colony formation, and induced G0/G1 phase arrest [127]. Moreover, studies on other cancer types showed both pro- and anti-tumorigenic effects of incretin-based drugs. Using real-world data Wang et al. detected a differential risk association between GLP-1RAs treatment and cancers. They showed that GLP-1RAs were associated with lower risks of prostate, lung, and colon cancer, but a higher risk of thyroid cancer [128]. These results strongly indicated that complex mechanisms may involve in the interaction between incretin-based drugs and cancer.

As with other cancer types, research on incretin-based drugs and BTC began more than a decade ago. In 2007, Marzioni and colleagues found that the GLP-1receptor is expressed in cholangiocytes and will be up-regulated in the course of cholestasis. GLP-1 increases cholangiocyte growth both in vitro and in vivo through the PI3K/cAMP/PKA-Ca2+-CamKII pathway instead of the classical proliferative MAPK-ERK1/2-PKCα pathways. The proliferative effects can be blunted by GLP-1RAs [128]. Later in 2009, they found that exendin-4, a 39-amino acid GLP-1 analog, prevents cholangiocyte apoptosis both in vitro and in vivo by counteracting the activation of the mitochondrial pathway of apoptosis [129]. In 2014, Chen et al. showed that the GLP-1 receptor protein expression was upregulated in tumor tissue samples of patients with ICC and associated with lymph node metastasis [130]. These studies initially raised concerns regarding a potential association between incretin-based drugs and increased risk of BTC.

In 2016, the Liraglutide Effect and Action in Diabetes: Evaluation of Cardiovascular Outcome Results (LEADER) trial firstly reported the association of liraglutide with BTC, yielding an increased hazard of 1.62 (95%CI, 0.67–3.90) [131]. This study failed to clarify the relationship between saxagliptin and BTC because of the wide confidence interval. In 2018, an observational study conducted by Abrahami et al. attracted widespread and intense interest. Using data from the UK Clinical Practice Research Datalink (CPRD), the authors found that the use of DDP-4i was associated with a 77% increased hazard of CCA (DDP-4i HR, 1.77; 95% CI, 1.04–3.01) and the use of GLP-1 RAs was associated with an increased hazard with a wide confidence interval (GLP-1 RAs HR, 1.97; 95%CI, 0.83–4.66) [17]. However, although 154,162 patients were included in this cohort, only 105 incident BTC events occurred, of which 27 events occurred in the DPP-4i group and 7 occurred in the GLP-1 RAs group. The small number of cases prevented this study from more convincible conclusions.

However, the results of different studies are inconsistent. In 2015, the Saxagliptin Assessment of Vascular Outcomes Recorded in Patients with Diabetes Mellitus (SAVOR)–Thrombolysis in Myocardial Infarction (TIMI) 53 trial firstly reported that the use of saxagliptin was associated with a lower hazard of hepatic and biliary neoplasm (0.75; 95% CI, 0.30–1.76) [132]. In 2020, a nested case-control study (744 cases vs. 2976 controls) showed that no increased odds ratio (OR) of CCA associated with the use of either DPP-4i (OR, 0.98; 95% CI, 0.75–1.29) or GLP-1 RAs (OR, 1.09; 95% CI, 0.63–1.89) [133]. Similarly, a nationwide study using data from three Scandinavian countries in 2021 showed that neither the use of DPP-4i nor GLP-1 RAs was associated with a significantly increased risk of CCA (DPP-4i HR, 1.15; 95% CI, 0.90–1.46; GLP-1 RAs HR, 1.25; 95% CI, 0.89–1.76) [134]. Different from a study using data from the CPRD, this study had a larger sample size (n = 585,876) and more BTC cases (n = 599), of which the DPP4i group included 346,485 patients with 350 BTC events occurred and the GLP-1 RAs group included 239,391 patients with 249 BTC events occurred. More importantly, although 25% of the patients with a long-term follow-up of more than 6 years (7.0 years in the DPP4i group and 6.9 years in the GLP-1 RAs group), no elevated risk was found. This indicated that lifelong use of incretin-based drugs might be safe for BTC in patients with T2DM. To resolve the controversy between different studies, He et al. published a comprehensive meta-analysis of randomized clinical trials in 2022. A total of 76 randomized control trials (RCTs) involving 103,371 patients were included. Although the results showed that GLP-1 RAs treatment was associated with increased risks of other gallbladder or biliary diseases, there was no increased relative risk (RR) of BTC (RR, 1.43; 95% CI, 0.80–2.56). Of note, only 25 cases of BTC were observed in GLP-1 RAs groups (25/31,010) and 15 in control groups (15/30,026) [16] (Results of epidemiological studies are summarized in Table 1).

### Concerns from supportive evidence: incretin-based drugs and biliary diseases

Despite the conflicting results, there is evidence to support the possible association of incretin-based drugs with an increased risk of BTC. As a complex disease, BTC is affected by many risk factors, including biliary tract diseases. Incretin-based drugs have been reported to disrupt biliary homeostasis, which has implications for the development of BTC.

Firstly, incretin-based drugs were associated with an increased risk of gallstone formation. Kellera et al. showed that GLP-1 RA exenatide significantly reduced CCK-induced gallbladder emptying compared to placebo in fasting healthy subjects [135]. The inhibition of gallbladder contractility leads to gallstone formation, which is an established risk factor for GBC. In a weight management trial of liraglutide, results showed that the use of liraglutide was associated with an increased risk of cholelithiasis [136] and consistent with results from other studies [132, 137]. The underlying mechanism for the slowed emptying of the gallbladder is that incretin-based drugs reduce smooth muscle tone in the gastrointestinal tract [138]. In addition, Rehfeld et al. showed that GLP-1 also attenuated gallbladder contractility by suppressing the secretion of cholecystokinin (CCK) after a meal in normal and diabetic subjects. The reduced CCK increased the risk of adverse gallbladder events during treatment with GLP-1-derived drugs [139]. Moreover, Smits et al. showed that liraglutide and sitagliptin induced changes in bile acids by altering the profile of the intestinal microbiome and increasing hepatic bile acid production, respectively [140]. These effects of incretin-based drugs all facilitate gallstone formation.

Secondly, incretin-based drugs are positively related to several inflammatory biliary diseases. Studies have shown that the use of GLP-1 RAs was associated with an increased risk of cholelithiasis, cholecystitis, and cholangitis [132, 141]. Consistently, in an updated meta-analysis of 76 randomized trials involving 103,371 patients, results showed that GLP-1 RAs treatment was associated with increased risks of cholelithiasis, cholecystitis, and biliary disease (including bile duct obstruction, stenosis, and stone; biliary colic, cyst, and fistula; biliary tract cancer; cholecystectomy, cholecystitis, and cholelithiasis; and cholangitis), and showed a dose-response relationship [16].

## Conclusion

Insulin and incretin-based drugs might be important risk factors for BTC in diabetic patients. It remains a challenge to elucidate their association due to the relatively low prevalence of BTC and the intricate molecular mechanisms involved. Furthermore, the current strategy of analysis seems to be problematic and will blur the truth. At present, most available studies did not take into account the heterogeneity of BTC. The current studies mixed all BTC types together for analysis or even included HCC in BTC for evaluation. This may be the main reason for the inconsistency between the results of different studies. For instance, if incretin-based drugs are significantly associated with an increased risk of ICC but not ECC and GBC, then studies that include more ICC cases are likely to conclude that incretin-based drugs are associated with an increased risk of BTC, and at one extreme, all BTC included are ICC. In contrast, those studies that include more ECC and GBC may conclude that incretin-based drugs are not associated with an increased risk of BTC. In consideration of the heterogeneity of BTC and the complex effects of different types of insulin and incretin-based drugs, the key to clarifying their relationship is to classify these drugs and BTC specifically into different subtypes during the investigation. We suggest that experimental studies in this field should classify the incretin-based drugs by specific pharmacological mechanisms and strictly identify different types of BTC by clinicopathological and molecular features. That is, a specific drug (e.g., exenatide) is paired with a specific BTC (e.g., ICC), rather than studying the relationship between GLP-1 and BTC in general. Particularly, separate experiments should be conducted to verify whether the conclusions drawn from one drug-cancer pair can be applied to another. This strategy is also applicable to the study of incretin-based drugs and other cancers, such as liraglutide-TNBC and liraglutide-MCF-7 as mentioned above. Furthermore, by taking advantage of electronic health record databases and conducting more large-scale and in-depth studies, more promising achievements can be obtained in the future.

## Data Availability

Not applicable.
